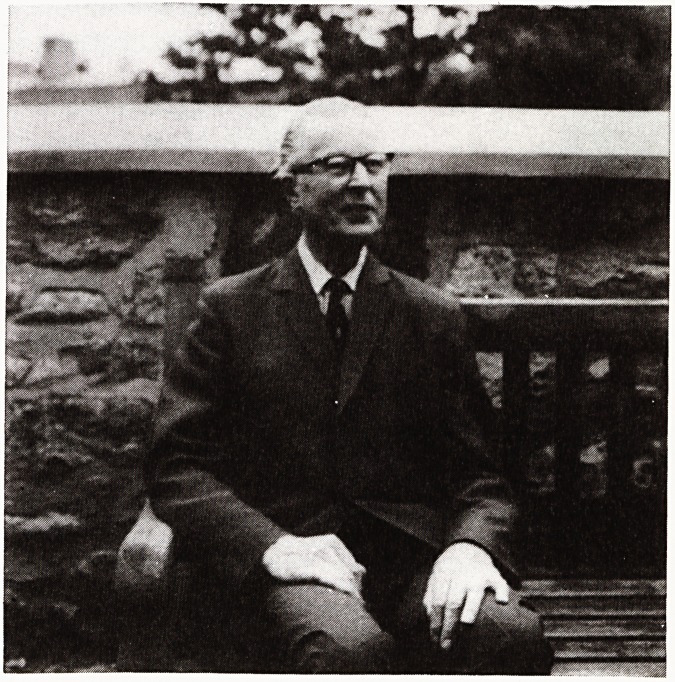# Mr Anthony Palin

**Published:** 1987-02

**Authors:** 


### Obituary

### Anthony Palin, BA, BM, BCH, FRCS, FRCSE

Mr Anthony Palin, formerly Consultant Ophthalmologist at Bristol Eye Hospital, died on 26th January 1986 after a long illness patiently borne.

Anthony Palin graduated BA at Oxford in 1930 and then entered St Thomas's Medical School, graduating in 1934. After post-graduate training and gaining the FRCSE he was appointed Consultant Ophthalmic Surgeon at Bristol Eye Hospital in 1937, a post he held until he retired in 1972. He performed the first corneal graft in Bristol in 1937. During the war he served in the Royal Air Force, seeing service in the Middle East and Germany and reaching the rank of Squadron Leader. On his return to Bristol after the war, he played a leading part in making the Hospital one of the best Eye Units in the country.

Tony was well-known and much respected both in Bristol and nationally. For over twenty years he was Head of the Department of Ophthalmology of Bristol University and was responsible for the organisation of teaching of medical students. For many years he was on the Council of the Section of Ophthalmology of the Royal Society of Medicine and also on the Council of the Faculty of Ophthalmologists, the body that organises the running of ophthalmology in this country. He also played a leading part in the South West Ophthalmological Society, becoming its President from 1959 until 1961.

Tony's colleagues appreciated his friendly co-operation , his courtesy and steadfastness. He had tremendous enthusiasm for his speciality and had the gift of affecting others with his enthusiasm. He played a big part in training over twenty Australian House Surgeons who are now eminent Consultants in different parts of Australia. He had great gifts of integrity and loyalty, and was loved by his patients for his kindness and skill.

He had a great sense of occasion and lived life to the full in every sphere. An outstanding marksman, he competed regularly at Bisley, and was a keen salmon fisher. He is survived by his wife Bobbie and two sons.

## Figures and Tables

**Figure f1:**